# Dr. Paschal’s Paper

**Published:** 1896-12

**Authors:** 


DR. PASCHALL'S PAPER.—In this issue of the JOURNAL will be found a paper by Dr. Frank Paschall, of San Antonio, which was read at the recent annual meeting of the West Texas District Medical Association. Dr. Paschall makes a plea in behalf of the unfortunate consumptives, and urges that the State shall make provision for the humane care of the indigent of that class.

Not alone does this subject appeal to our humanity, but in the interest of the public health, and of race integrity, it is the duty of the government to arrest the propagation of consumption by every legitimate means in its power. Not only should there be strict laws on the subject, with a penalty for their disregard or violation—for instance, as the author says—it should be made the duty of every physician having knowledge of a case of consumption to report it to the city health authorities, as is the case with other communicable diseases, in order that the danger, being known, may be avoided; and it should be the duty of hotel keepers to carry out strict sanitary measures, to be formulated by the city health authorities, for the purpose of preventing a spread to others; the duty of railway companies to provide separate sleepers for consumptives, or to disinfect every car after occupancy by a consumptive, but we go further, and insist that in the interest of posterity, and, ergo, of race integrity, consumptives shall be prohibited from marrying. If those in whom the disease has developed, or is developing; we might go further and say—those born of consumptive parents in whom the disease had developed in consequence of a hereditary predisposition,—do not have sufficient regard for the fate of their offspring, in case there should be any, and of the welfare of coming generations, to abstain from matrimony, they should be restrained by the State. Consumptive children should be prevented, as should all other defectives.

And we say this in full recognition of the fact, that the belief is now generally held, that consumption is not hereditary in the sense that insanity or syphilis is; and that the offspring of consumptive parents, i. e., those in whom there is known to be a hereditary taint—do not necessarily all develop consumption; that it depends upon environment to a great extent; yet no one will deny that many, if not most of them do; but there is transmitted a cachexia,—a weakness of vitality—a predisposition to the tubercular disease which renders them more likely to "catch" or acquire it, when brought into contact with it, than are those in whom no such taint exists. Else, why do we see, sometimes, whole families of children carried away with this disease, often before reaching puberty, or soon after? The saddest sight in life, is to see a young girl, born of consumptive parents, enter the state of matrimony, and after bringing into the world a progeny—more or less numerous—develop the disease and die; and then to see these children perish one by one. In Mississippi the writter knew of such a case in the family of a very eminent and able and distinguished physician. His wife inherited the blight. She bore him four sons before the disease developed, and carried her to a premature grave. And the sons—upon reaching the age of twenty or twenty-one, died as surely as if they had been—as they were, indeed, fated. They all studied medicine. But one lived to graduate, and on him his devoted father had centered his fondest—all his hopes. Alas, the ink on his diploma was scarcely dry before the fate of his mother and brothers overtook him. The doctor had, upon the death of his wife, married again, and had a family of healthy children. I can see his face now, as I saw him last;—he was the saddest man I ever saw, as he talked to me of his hopes for his sons, and how they had—one after another disappointed him, till his last one was gone, just as he had hoped to see him enter the profession to which his father was so devoted.

Dr. Paschall touches upon another point apropos to the development—or otherwise—of the predisposition, by environment: the management of delicate children in school. It is a most important subject, and one which should be discussed, and the people educated upon it—not only the point here mentioned—for reform in the sanitation of schools, school rooms and management of pupils is notoriously demanded, and teachers should be educated first into the requirements; but the whole subject of consumption, especially its mode of spreading and dissemination, should be written up in the daily press, that people may learn to appreciate the danger and avoid it. And as public sentiment is not yet ripe for such restrictive measures as quarantine, the people amongst whom the disease occurs, and the traveling public, and hotel men, hack owners, street car companies and sleeping car companies ought to understand that there is danger in the air, when a consumptive is permitted to cough and spit at random; and parents should know that kissing of a consumptive is dangerous to the other members of the family; that a consumptive should not sleep with a well person, nor in the same room with one. And knowing these things, self-interest, if no higher motive, will prompt them to put into execution measures of prevention.

Dr. Paschall's paper is timely and sensible. It is to be hoped that the legislature may be induced to make some provisions for the indigent consumptives, as suggested by Dr. Paschall, and also to enact statutes looking to the preventive of a spread of the disease on railways and in other public places. The latter function is really the duty of a State board of health, but in Texas we have no State board of health; and the State health officer is powerless, as no facilities are afforded him for the carrying out of any regulations he might formulate on the subject other than of quarantine. He holds views in accordance with the foregoing, and a short while ago issued a circular letter to local health officers, urging action in each locality as to the regulation of hotels and local conveyances in their relation to the contagion of consumption—which circular letter was published in the JOURNAL at the time; but as stated, he is powerless to compel it.

